# Unveiling the culprit: the fusobacterium lineage that populates colorectal cancer

**DOI:** 10.1038/s41392-024-01844-x

**Published:** 2024-05-08

**Authors:** Johannes Betge, Matthias P. Ebert

**Affiliations:** 1 Department of Medicine II, Medical Faculty Mannheim, Heidelberg University, Mannheim, Germany; 2 Junior Clinical Cooperation Unit Translational Gastrointestinal Oncology and Preclinical Models, German Cancer Research Center (DKFZ), Heidelberg, Germany; 3 DKFZ Hector Cancer Institute at University Medical Center, Mannheim, Germany; 4 MMPU Microbiota Drug Metabolism and Cancer Therapy, European Molecular Biology Laboratory, Heidelberg, Germany

**Keywords:** Cancer, Cancer

**Fig. 1:**
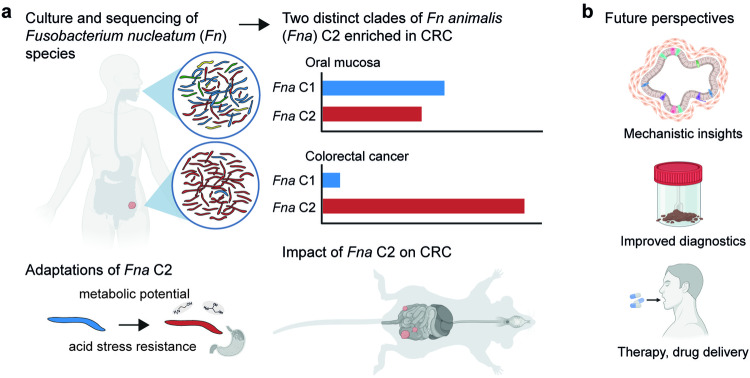
*Fusobacterium nucleatum animalis* clade 2 (*Fna* C2) is enriched in colorectal cancer (CRC) and contributes to tumor progression. **a** Schematic overview of the approach and major findings presented by Zepeda-Rivera et al.^1^: Genomes and methylomes of *Fn* cultures from oral mucosa of healthy individuals and CRCs were analyzed to discover that a specific clade of *Fna* is enriched in CRC. This clade (*Fna* C2) has specific enhanced metabolic traits and improved resistance to acid-related stress, promotes tumor progression as well as alterations in intestinal metabolism in mice. It was also proven to be the dominating *Fn* strain in metagenomics datasets of CRC. **b** The findings direct the focus of future studies on the role of *Fn* in CRC on this specific clade and may thereby prompt novel developments in diagnostics and therapy of CRC. The figure was adapted from the publication by Zepeda-Rivera et al.^1^ and created with BioRender.com

In a recent study published in *Nature*, Zepeda-Rivera et al.^1^ discovered that one distinct clade of *Fusobacterium nucleatum animalis* (*Fna*) dominates the colorectal cancer (CRC) tumor niche. The work unravels a more precise understanding of a major bacterial species associated with CRC progression, thereby establishing a new focus for future studies, which may aid to develop improved microbiome-based diagnostics and treatments (Fig. 1).

CRC is one of the most frequent human cancers and second leading cause for cancer related deaths in the US, even though it could be cured by detecting and removing early-stage disease or its benign precursors. Early detection is thus a major clinical challenge, as is the treatment of advanced and metastasized disease. The emergence of next-generation sequencing and metagenomic studies have led to a vast increase of data associating different microbiota and microbial communities with CRC. However, precise understanding of distinct species’ contributions to tumor progression and underlying mechanisms is largely lacking. This is currently leading to a re-emergence of classical microbial culture approaches for functional studies. *Fusobacterium nucleatum* (*Fn*) usually occurs in the healthy human oral flora. It is rarely found in the gastrointestinal tract; however, large-scale sequencing-based analyses have repeatedly found it to be associated with CRC.^2^ Moreover, high abundance of *Fn* within CRCs has been associated with metastatic disease and shorter survival rates of patients, prompting the question of a causal link to tumorigenesis and tumor progression. Several subspecies and variations of *Fn* strains exist and up until now, it has been unknown which were most important to CRC.

Zepeda-Rivera et al. analyzed the complete genomes and methylomes of 65 *Fn* cultures from patients with CRC and 81 *Fn* strains from oral mucosa of healthy individuals. Of the different *Fn* subspecies, only *Fna* was enriched in CRC cultures. Surprisingly, *Fna*, which was previously believed to represent a uniform subspecies, branched out into two distinct clades: The first, *Fna* C1, restricted to oral mucosa and a second, *Fna* C2, dominating the CRC niche.

How can *Fna* C*2* be highly prevalent in CRC, while the C1 clade, as other *Fn*, are mainly restricted to the oral cavity? The authors showed that *Fna* C1 and C2 clades are genetically and epigenetically distinct and they identified factors consistent with increased potential to colonize the gastrointestinal tract, in particular enhanced metabolic capabilities for intestinal metabolites. Additionally, differences in resistance towards *pH* related stress between C1 and C2 were experimentally shown, which may contribute to *Fna* C2 abilities to transit through the gastrointestinal tract.

Colonization of CRCs by *Fn* has repeatedly been shown, however, the detailed roles and mechanisms behind this are largely unclear. The discussed study used a DSS-colitis promoted APC^min+/−^ mouse model of CRC to functionally prove a driving role of *Fna* C2. Indeed, after oral gavage of *Fna* C2, mice had significantly higher numbers of large intestinal adenomas compared to *Fna* C1 and vehicle control. Metabolic analyses of the animals revealed hints of *Fna* C2 to promote oncogenic conditions in the intestine, linked to glutathione metabolism, oxidative stress, and inflammatory pathways. Total numbers of tumors were small, though, pointing to a role of *Fna* C2 in tumor progression rather than initiation. This matches with previous observations associating *Fn* to metastatic disease and poorer patient outcomes. *Fn* is also associated with right-sided tumors and microsatellite instability,^3^ exact mechanisms behind these associations remain to be elucidated.

The group also analyzed the prevalence of *Fna* species in human tissue and stool, i.e. without previous culturing of the bacteria - a prerequisite for a potential translation of the findings to clinical diagnostics. Indeed, in 16s rRNA sequencing studies of tumor-adjacent-normal-tissue pairs (*N* = 116 CRCs with 62 normal tissues) and a second CRC cohort (*N* = 86), only *Fna* C2 was enriched in tumor tissue. Re-analyzing published stool metagenomic datasets of 596 patients with CRC and 616 healthy persons, they found *Fna* in 29% of CRC patients and 5% of healthy individuals. Both *Fna* clades were found in CRC stool samples, but *Fna* C2 was more prevalent and was the only *Fn* subgroup significantly enriched. Collectively, these findings confirm that *Fna* C2 is the primary lineage of *Fn* to affect CRC.

The findings open new avenues of research. Generally, experimental methods and analyses of bacterial virulence factors in the carcinogenic process and in tumor progression have begun to emerge in recent years. For instance, colibactin producing E. coli species have recently been shown to induce distinct mutational signatures associated with CRC, using luminal injection techniques with intestinal organoid models.^4^ It remains to be shown whether and how *Fn* interacts with CRC cells in detail, yet the present work by Zepeda-Rivera et al. allows future mechanistic studies to focus on the *Fna* C2 clade as the highly prevalent and virulent subgroup of *Fn* in CRC. Further experimental studies may seek to define which genes and pathways are involved and how interactions with tumor cells, microenvironmental cells, especially the immune system, or bacterial communities enable *Fna* C2 to contribute to cancer progression and therapy response in the different colorectal cancer subtypes. The specific enrichment of *Fna* C2 may also prompt translational research aiming at more precise microbiome-based diagnostics and treatments. For instance, stool-based early detection methods may benefit from specifically testing for *Fna* C2 amongst other microbiome and non-microbiome related markers to increase specificity. Further studies will also be necessary to assess enrichment and specificity of *Fna* C2 to colorectal adenomas and in non-western populations. Additionally, the detection of *Fna* in both primary tumors and in metastases in previous studies,^5^ may suggest to explore strategies using *Fna* C2 based therapeutic targeting, drug delivery, or to exploit its putative role in differential immune regulation in microsatellite stable and instable CRC subtypes.^3^

Overall, the work identifies the specific *Fn* strain that populates the CRC niche and provides the first mechanistic data on a role of *Fna* C2 in CRC progression. This work directs the focus of future studies on this specific clade and may thereby prompt novel developments in the diagnostics and therapy of CRC.
